# Characterizing Pathways of Non-oral Prescription Stimulant Non-medical Use Among Adults Recruited From Reddit

**DOI:** 10.3389/fpsyt.2020.631792

**Published:** 2021-01-25

**Authors:** Suzanne K. Vosburg, Rebekkah S. Robbins, Kevin M. Antshel, Stephen V. Faraone, Jody L. Green

**Affiliations:** ^1^Inflexxion, An IBH Company, Costa Mesa, CA, United States; ^2^Department of Psychology, Syracuse University, Syracuse, NY, United States; ^3^Department of Psychiatry, State University of New York (SUNY) Upstate Medical University, Syracuse, NY, United States

**Keywords:** ADHD, prescription stimulants, prescription stimulant non-medical use, prescription stimulant non-oral use, transitions

## Abstract

**Objective:** Prescription stimulant non-medical use (NMU) is a national predicament. While the risks of prescription stimulant NMU have been considered, less is known about non-oral use. To focus on this gap, a sample of adults with non-oral prescription stimulant NMU within the last 5-years was recruited. The purpose of the present study was to characterize the pathways and substance transitions associated with prescription stimulant NMU and non-oral prescription stimulant NMU in this unique sample of adults.

**Methods:** Adults (*n* = 225) reporting non-oral prescription stimulant NMU within the last 5 years were recruited to complete an online survey by banner ads placed on the Reddit website between February and September 2019. After completion of the survey, a second study consisting of an in-depth telephone interview was conducted with 23 participants: interviews took place between July and September 2019. Data reported here include substance, route of administration and class transitions, as well as qualitative data from the interviews.

**Results:** Approximately 1 in 5 began their substance use trajectory with prescription stimulants (19.1%). Other than marijuana, most exposures to illicit substances occurred after both initial prescription stimulant NMU and initial non-oral prescription stimulant NMU. The most frequently reported route of administration transition was from oral use to snorting (*n* = 158, 70.2%), however, other route of administration transitions included oral use to injection drug use (*n* = 14, 6%). In-depth interviews elaborated upon these transitions and indicated that prescription stimulant NMU was consequential to substance use pathways.

**Conclusions:** Oral prescription stimulant NMU was a precursor to non-oral prescription stimulant NMU. Non-oral prescription stimulant NMU was a precursor to illicit substance use, suggesting that prescription stimulant NMU impacts substance use pathways and revealing opportunities for intervention.

## Introduction

Prescription stimulant non-medical use (NMU) is a persistent, national dilemma (1–4). Medications containing amphetamines (e.g., Adderall, Vyvanse) or methylphenidate (e.g., Ritalin, Concerta, Focalin) are considered the most widely prescribed stimulants in the United States (5, 6), and are regarded as the most efficacious drugs in the management of ADHD symptomatology (7). These medications lead to regional elevations in brain dopamine (8–10) rendering them as potential candidates for non-medical use (NMU) and diversion (8, 11–17).

Non-oral NMU of prescription stimulants—use that involves alternate routes of administration including intranasal or intravenous routes—has been reported in adolescents (18–21), college students (22–25), and adults (26–28). While the physical and psychiatric risks as well as mortality associated with prescription stimulant NMU have been considered (29–34), less is known about *non-oral* prescription stimulant NMU, which can include adverse physical outcomes, such as toxicity or tissue damage (35–40), and adverse mental health outcomes, such as anxiety or depression (41) or even psychosis (33).

The transition from oral to non-oral NMU of prescription stimulants is not yet well-documented. However, there is an analogous framework to be found in the study of opioid use pathways and transitions (42–47). For example, an opioid class transition is the precursory use of prescription opioids before the use of heroin (48–51). This class transition has been related to age, availability/supply, drug quality, and surrounding environments (52–56). An opioid route of administration (ROA) transition (e.g., swallowing intact tablets then transitioning to crushing the tablet and snorting or injecting) is thought to occur when individuals develop tolerance to effects of a substance and desire stronger effects, more rapid onset of effects, or a more economical way of achieving them (42, 43, 57). Initial route of administration can also affect subsequent ROA choices and overall pattern of drug use (58, 59).

Substance transitions are broadly captured by polysubstance use or substitutions, i.e., when one substance is not available, another is used (54, 55). In addition to switching from one substance or route of administration to another, where previously used substances or routes of administration are no longer used, a substance transition can also mean that new substances (and by extension routes of administration) are simply added to the current repertoire. Which is to say, substance use trajectories are not necessarily linear.

Prescription stimulant NMU class transitions would include prescription stimulant NMU leading to illicit stimulant use, such as cocaine or non-prescription methamphetamine. Prescription stimulant NMU route of administration transitions may include moving between oral and non-oral routes of administration (26, 60). Prescription stimulant substance transitions would involve moving on to other substances after prescription stimulant NMU, a trajectory that is possible among individuals with polysubstance use; a characteristic reported in individuals endorsing prescription stimulant NMU (61–66).

The purpose of this paper is to inform future hypothesis generation by identifying substance transitions, route of administration transitions and class transitions that were reported by a sample of adults who undertook prescription stimulant non-oral NMU within the last 5 years. This convenience sample was recruited from Reddit. The study employed a mixed-method design incorporating quantitative and qualitative data analyses.

## Materials and Methods

For purposes of this study, NMU included ANY of the following: (1) use for any reason, even once, without your own prescription, (2) use in ways other than prescribed (such as taking more than prescribed, more often than prescribed, or for any other reason or way than prescribed), and (3) use for the feeling or experience the medication caused (such as a feeling of being high, enhancement of other drugs, prevention or treatment of withdrawal symptoms, or other feelings).

### Procedure

Advertisement banners appeared on Reddit (http://www.reddit.com) from February through September 2019 for purposes of study recruitment. Banner ads stated that adults (aged 18 years or older) who were English-speaking, had personal experience of non-oral prescription stimulant NMU within the last 5 years, able to give informed consent and were interested in taking an online survey could click on an embedded survey link. The link took them to an online web survey hosting site (YouGov) for survey completion.

Respondents were first asked to provide written, informed consent to participate. Survey completion took ~10–15 min; participation could be stopped at any time. Respondents who completed the online survey were compensated with a $20 e-gift card upon completion. Compensation was managed by a third party to reduce privacy concerns.

After completion of the online survey, individuals were presented with the option to participate in a more in-depth, semi-structured, follow-up telephone interview to further elucidate their experience with prescription stimulant medications. If respondents agreed to complete the follow-up interview, a designated time was confirmed for interviewers to call them.

Verbal informed consent was acquired at the beginning of each telephone interview; interviews were audio recorded with the participant's permission. Interviewers used an interview guide with open-ended questions that were designed to elicit in-depth responses about participants' substance use. Participants were compensated an additional $25 for their participation, and again, compensation was managed by a third party. After the interviews were transcribed, audio recordings were deleted. This study was approved by the New England Institutional Review Board (NEIRB): 120180324 #137173.0.

### Survey and Follow-Up Interview Description

The online survey consisted of four sections: demographics, medical history, history of prescription medication NMU, and history of illicit substance use with 15 broad topic areas. To ensure reliable and accurate prescription medication identification, product images were displayed and participants were asked to indicate those they had used. To determine use characteristics, such as motivations for use, respondents were asked to select among pre-determined, categorical responses, however, “Other” with a write-in response area was always an option. Skip logic was employed so that number of survey items varied depending on number of prescription medications that were identified as having been used non-medically. Follow-up interviews were ~1 h in duration. At the outset, after informed consent had been provided, participants were asked to confirm the use of prescription stimulants that had been described in their online survey. After confirmation that this information was, indeed, accurate, participants were asked a series of open-ended questions detailing their use, a series of questions about their patterns and pathways to substance use and a series of questions about their experiences regarding manipulation deterrent formulations. For purposes of this report, the patterns and pathways of substance use are of interest.

### Data Handling and Analyses

Survey data were uploaded to and stored on a password protected Inflexxion server that was only accessible by authorized study personnel. The server resided in a climate-controlled, locked facility with nightly backups. Audio recordings were destroyed after a transcription was made from the recordings.

All analyses were carried out using SAS Enterprise Guide Version 7.1 (Cary, NC). Self-reported responses to quantitative survey questions were analyzed with descriptive frequency analyses or ordered categorically. Interviews from the follow-up interview were transcribed using an AI transcription platform. Interview results were categorically combined and summarized on an individual level.

## Results

Between February and September 2019, 225 participants were recruited from Reddit and completed the online survey. The sample was primarily male (86.2%), not of Spanish, Latino or Hispanic origin or descent (92.4%), White (78.2%), and 25+ years of age (52.0%), with some amount of college education (81.3%). Most were single (67.6%) and working full-time or part-time (60.4%) with an annual family income between $30,000 and $99,999 (49.3%). The majority reported at least one psychiatric diagnosis in their lifetime (55.1%), most of which were depression (32.9%), anxiety (28.9%), or ADHD (27.6%). Participants were on average, 18.7 (±3.7) years of age when they first initiated prescription stimulant NMU.

Twenty-three participants from the original sample (10.2%) were contacted and completed follow-up interviews, between July and September 2019. This subset of participants was primarily male (*n* = 20, 87.0%) and 28.2 (range 19–36) years of age. Almost half had obtained prescription stimulants through a healthcare provider (*n* = 11, 47.8%) and almost all had obtained prescription stimulants through diversion (*n* = 22, 95.7%). Qualitative follow-up interview results are presented in the latter half of this paper.

Among the full sample, lifetime prescription stimulant NMU included amphetamine (*n* = 209, 92.9%) or methylphenidate (*n* = 103, 45.8%). Other reported lifetime prescription drug use (with or without prescriptions, for any reason) included opioids (44.4%), sedatives (40.9%), muscle relaxants (24.4%), sleep aids (23.6%), and/or diet aids/appetite suppressants (8.0%). In addition to lifetime prescription stimulant NMU, participants reported prescription stimulant NMU within the past year (*n* = 171, 76.0%) and/or within the past month (*n* = 81, 36.0%), while some reported their last use as being longer than a year ago (*n* = 54, 24.0%). Past year and past 30-days prescription drug use were also reported, although to a lesser degree.

### Substance Transitions, Stimulant Class Transitions

**Figure 1:**
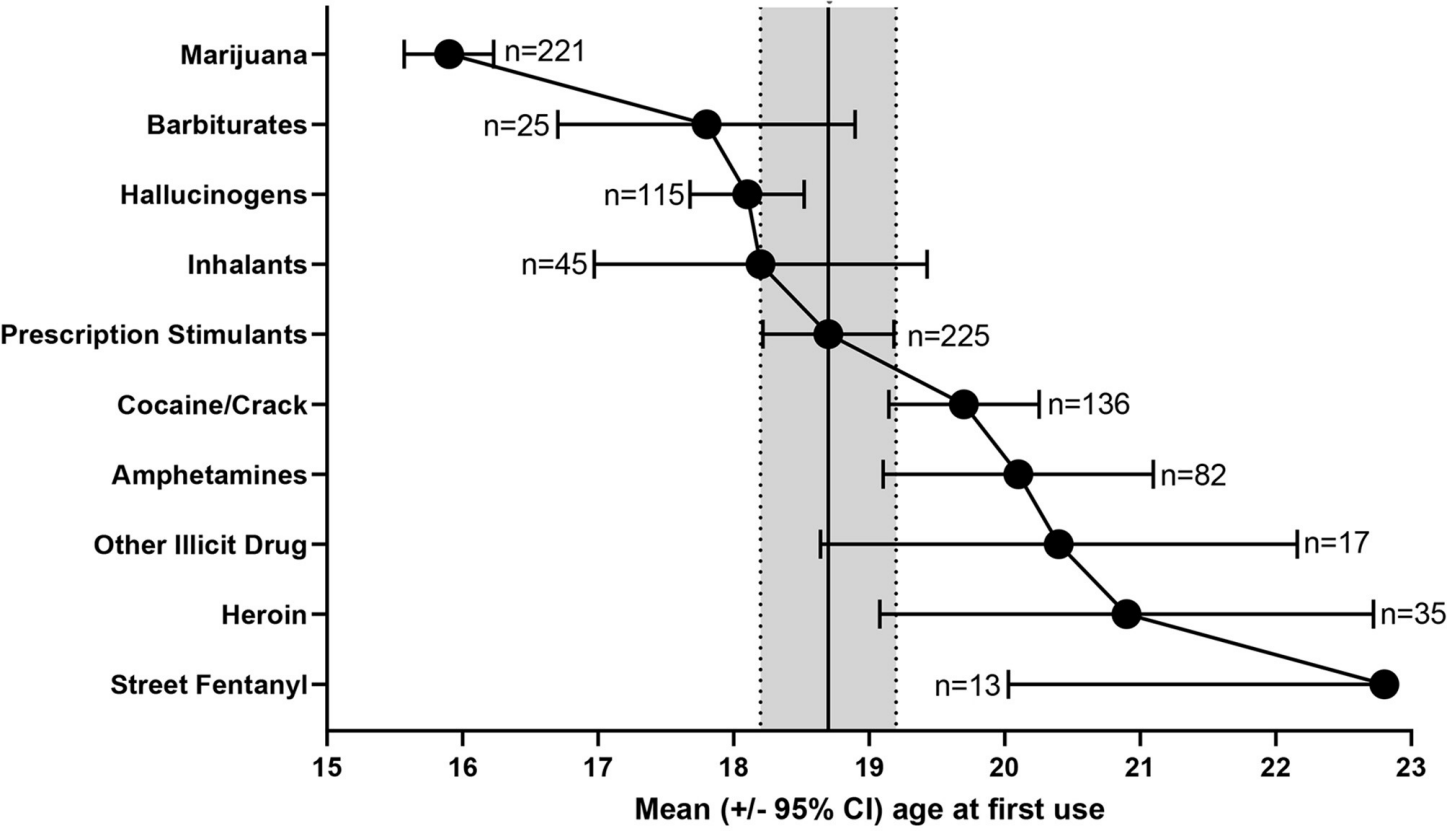
Mean age ± 95% CI of initial prescription stimulant NMU and illicit substance use among individuals reporting non-oral prescription stimulant NMU (*n* = 225).

Slightly more than 3 of 4 began their substance use pathway with marijuana (*n* = 173, 76.9%), while almost 1 in 5 began this pathway with prescription stimulants (*n* = 43, 19.1%). Figure 1 summarizes the mean age and 95% Confidence Interval (95% CI) at which various illicit substances were initially used by the sample in relation to prescription stimulants. Figure 1 reveals that marijuana use began earlier than other illicit drug use (95% CIs do not cross with any other substance). Otherwise, the 95% CIs of the means overlap, revealing that the mean age of initiation did not substantially differ between prescription stimulant NMU and the use of remaining illicit substances, other than illicit fentanyl, that was initiated at a significantly later age than prescription stimulants.

#### Stimulant Class Transitions

Even though the differences were not statistically significant, chronologically, prescription stimulant NMU preceded illicit stimulant use (age of first prescription stimulant NMU: 18.7 ± 3.7 years, age of first cocaine/crack use: 19.7 ± 3.3 years, age of first illicit amphetamine use: 20.1 ± 4.6 years). Three respondents (1.3%) reported using only prescription stimulants non-medically without any illicit substance use.

Table 1 examines substance use patterns by mentions of initial exposure with substance and route of administration as one instance, hence more than one instance may be reported by one individual and frequencies may be greater than the sample size. For example, there were 259 mentions of marijuana use in this data set. Of these, *n* = 38 (14.7%) mentions were the first oral use of marijuana and *n* = 221 (85.3%) were the first non-oral use of marijuana. Table 1, A columns summarizes how other than marijuana, most exposures to illicit substances occurred after the first episode of prescription stimulant NMU.

**Table 1:**
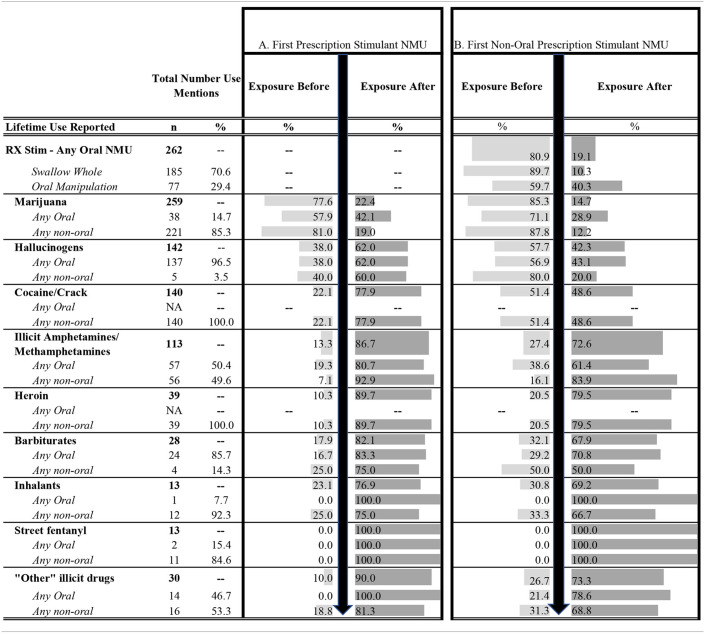
Substance transitions: lifetime illicit substance use in relation to prescription stimulant NMU organized by whether initial substance exposure took place before or after the first episode of prescription stimulant NMU (A) or before or after the first episode of prescription stimulant non-oral NMU (B).

*^a^Analysis includes all oral and non-oral routes of administration reported for substances ever used by respondents reporting lifetime non-oral NMU of prescription stimulants. ‘Other’ routes reported for substances listed are not included*.

*^b^Initiation before or after prescription stimulant NMU initiation was determined by the respondents self-reported order of first time a route route/substance combination was used*.

*^c^Any oral includes swallowing whole or chewing or dissolving before swallowing*.

*^d^Oral manipulation includes chewing or dissolving before swallowing*.

*^e^Any non-oral includes snorting, smoking, or injecting*.

Specifically, 77.6% of marijuana exposures occurred prior to initiation of prescription stimulant NMU, while 22.4% occurred after the first prescription stimulant NMU. Further, 57.9% oral exposures were before and 42.1% exposures were after first prescription stimulant NMU, while 81.0% non-oral exposures were before and 19.0% non-oral exposures were after first prescription stimulant NMU. Representing a potential class transition, 77.9% cocaine exposures and 86.7% of illicit amphetamine or methamphetamine exposures occurred after prescription stimulant NMU. This pattern was also found for other initial illicit substance exposures, for example, 62.0% of hallucinogen exposures, 89.7% heroin exposures, 82.1% of barbiturate exposures, etc., occurred after prescription stimulant NMU. This pattern held whether the illicit substance use was oral or non-oral (Table 1, A columns).

Table 1, B columns reveals that 80.9% of oral prescription stimulant NMU occurred prior to non-oral prescription stimulant NMU. In addition, most marijuana (85.3%) and hallucinogen (57.7%) exposures occurred prior to non-oral prescription stimulant NMU. Representing a potential class transition, cocaine exposures were almost evenly divided with slightly more exposures (51.4%) occurring before initial non-oral prescription stimulant NMU, while most exposures to illicit amphetamines occurred after initial non-oral prescription stimulant NMU. Most exposures to the remainder of substances (heroin, barbiturates, inhalants, etc.) also occurred after initial non-oral prescription stimulant NMU; all exposures to fentanyl occurred after initial prescription stimulant non-oral NMU.

#### Substance Transitions: Prescription Stimulant NMU to Prescription Opioid NMU

In addition to prescription stimulant NMU and illicit drug use combinations, ~38.2% of the sample (*n* = 86, 79.1% male, 20.9% female) reported lifetime prescription opioid NMU. Table 2 summarizes that a greater proportion of those with a history of prescription opioid NMU than without reported using prescription stimulants via any oral route (91.9 vs. 81.3%) that included cutting and breaking their prescription stimulants into smaller pieces before swallowing (31.4 vs. 5.8%), as well as chewing them before swallowing (20.9 vs. 5.8%). Among non-oral routes of administration, a greater proportion of those with a history of prescription opioid NMU than without reported smoking (8.1 vs. 0.7%) or injecting (10.5 vs. 3.6%) prescription stimulants.

**Table 2:** Routes of administration for prescription stimulant NMU with or without lifetime prescription opioid NMU.

**Characteristics of sample**	**Prescription stimulant non-oral NMU with prescription opioid NMU (*****n*** **= 86)**		**Prescription stimulant non-oral NMU without prescription opioid NMU (*****n*** **= 139)**	
	***n***	**%**	***n***	**%**
Any oral route	79	91.9	113	81.3
Swallowed whole	74	86.1	111	79.9
Cut or broke into smaller pieces then swallowed	27	31.4	8	5.8
Chewed in mouth then swallowed	18	20.9	8	5.8
Dissolved in liquid then swallowed	9	10.5	7	5.0
Any Non-oral route	86	100.0	139	100.0
Snorted	85	98.8	138	99.3
Smoked	7	8.1	1	0.7
Injected	9	10.5	5	3.6

### Prescription Stimulant Route of Administration Transitions

**Figure 2:**
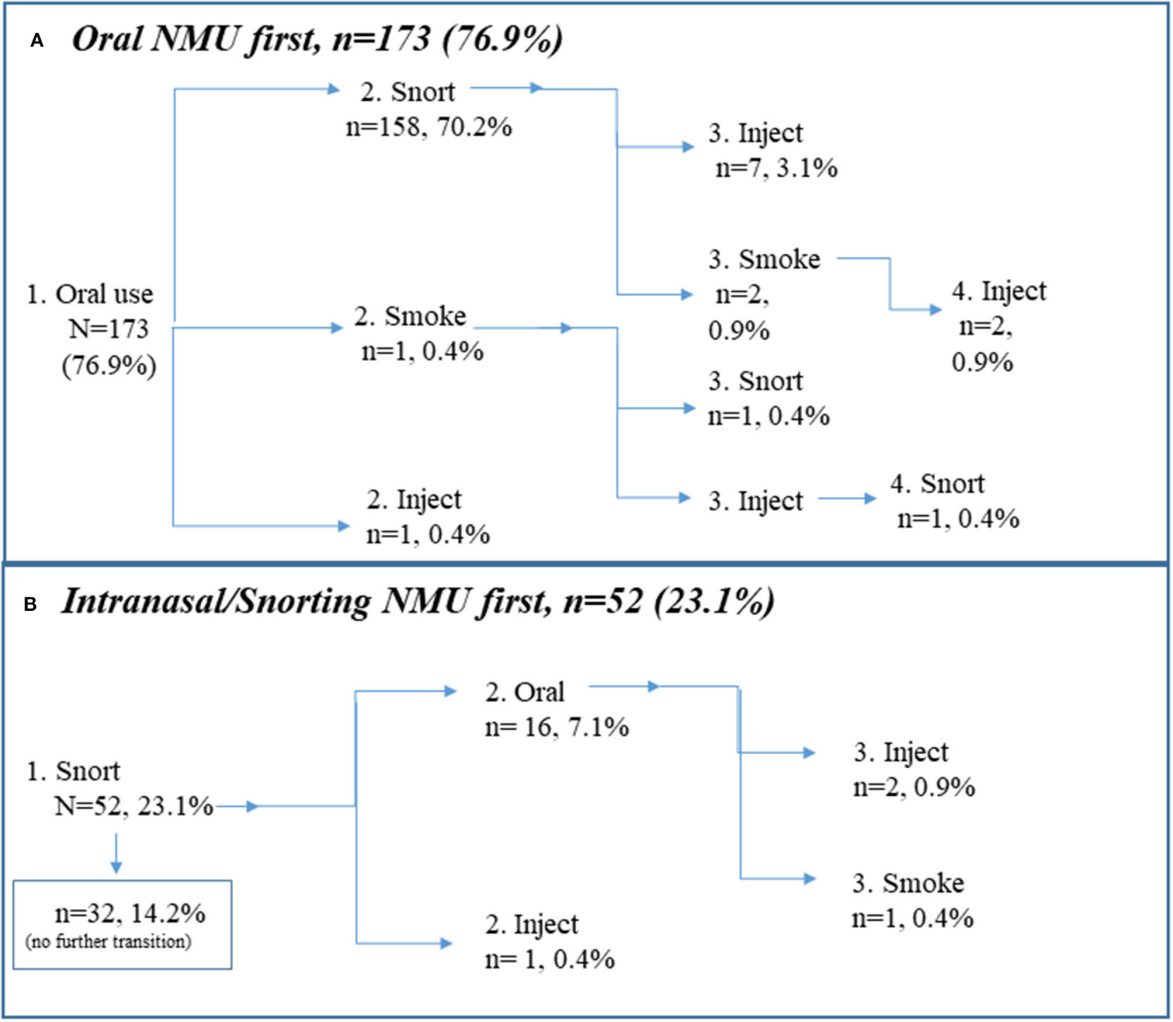
Route of administration transitions for prescription stimulants. Note that linear depiction is only for purposes of illustration. Routes of administration vary as necessitated by access and situation. **(A)** Oral prescription stimulant use first (*n* = 173, 76.9%), **(B)** Intranasal prescription stimulant use first (*n* = 52, 23.1%).

Figure 2 depicts transitions among the various routes of administration employed for prescription stimulant NMU among individuals who reported non-oral NMU experience. The first route of administration reported was either oral (swallowing: *n* = 173, 76.9%) or intranasal/snorting (snorting: *n* = 52, 23.1%). Figure 2A depicts that the majority, 70.2% (*n* = 158), snorted after initial oral use, and did not transition beyond that experience. Other patterns along this pathway involved oral->snort->inject (*n* = 7, 3.1%), oral->snort->smoke (*n* = 2, 0.9%), or oral->snort->smoke->inject (*n* = 2, 0.9%). Remaining pathways involved oral->smoke (1%), oral->smoke->snort (*n* = 1), or oral->smoke->inject->snort (*n* = 1). One individual reported oral use and transitioning to prescription stimulant injection.

Figure 2B depicts that the remainder, 23.1% (*n* = 52), snorted prescription stimulants for NMU first. Of those, *n* = 32 (14.2% of the entire sample/61.5% of those who snorted prescription stimulants for NMU first) did not transition to another route of administration. Other patterns involved snorting to oral use (*n* = 16, 7.1%) snort->oral->inject (*n* = 2, 0.9%), or snort->oral->smoke (*n* = 1). One individual reported snorting and then transitioning to prescription stimulant injection.

### Follow-Up Interview Depicting Motivations and Transitions

Table 3 summarizes motivations, positive and negative effects of prescription stimulant NMU endorsed by participants who completed the follow-up qualitative study (*n* = 23). Prescription Stimulant NMU was undertaken to enhance school work or work performance (*n* = 16, 69.6%) for recreational substance use, such as to get high or party (*n* = 16, 69.6%), or for the desire to treat ADHD when the regular dose was not achieving the effect (*n* = 11, 47.8%). Main positive effects of prescription stimulants included feeling Alert (*n* = 18, 78.3%), Stimulated (*n* = 8, 34.8%), or happy (*n* = 7, 30.4%), whereas main negative effects included feeling tired (*n* = 17, 73.9%), having a decreased appetite (*n* = 13, 56.5%), or feeling anxious (*n* = 9, 39.1%).

**Table 3:** Motivations, positive and negative effects of prescription stimulant NMU among follow-up interview respondents^*^.

**Motivations**	***n***	**%**
To enhance school or work performance	16	69.6
Recreational use. To get high/party	16	69.6
To treat ADHD when regular dose wasn't achieving effect	11	47.8
For energy or to stay up	10	43.5
To enhance effect of other drugs	4	17.4
To improve mood, self-medicate for depression, anxiety	3	13.0
To prevent or treat withdrawal symptoms	1	4.3
**Positive effects**		
Alert, focused, awake, better concentration	18	78.3
High, good buzz, stimulated	8	34.8
Happy, elevated mood, less depressed, less anxious)	7	30.4
Calm	6	26.1
Productive	5	21.7
Social	5	21.7
Energetic	4	17.4
**Negative effects**		
Insomnia, tired, exhausted	17	73.9
Decreased appetite	13	56.5
Anxiety	9	39.1
Strung out, restless, antsy	6	26.1
Cold sweats, feel terrible after crashing/coming down off stimulants	5	21.7
Rapid heartrate/palpitations	4	17.4
Paranoia and social anxiety	4	17.4
Tense, achy, headaches	3	13.0
Nausea	3	13.0
Depression	3	13.0
Irritable, impatient	3	13.0

* *Responses are not mutually exclusive and do not sum to 100%*.

Almost all respondents reported oral prescription stimulant NMU (*n* = 22, 95.7%), and most reported that their first episode of prescription stimulant NMU was oral (*n* = 20, 87.3%). Similarly, almost all reported snorting prescription stimulants (*n* = 22, 95.7%) but few indicated that their first episode of NMU involved snorting (*n* = 3, 13.0%). Thirteen percent (*n* = 3) reported injecting prescription stimulants. Participants did not typically endorse snorting or smoking for performance enhancement purposes. The primary motivation for snorting or injecting was to achieve a faster impact of the drug (snorting, *n* = 21, 95.4%; injecting, *n* = 2, 66.7%). Additional motivations for snorting included curiosity/others were doing it (*n* = 12, 54.5%), a friend was doing it (*n* = 3, 13.6%) or the person liked it and thought it was cool (*n* = 6, 27.3%). Additional motivations for injecting were the ritual (*n* = 1) or curiosity (*n* = 1). Table 4 summarizes representative responses endorsing various motivations to use prescription stimulants via snorting or injecting routes of administration.

**Table 4:** Motivations for snorting or injecting prescription stimulants for NMU (*n* = 23).

**INTRANASAL**
**Faster impact**
• I knew the effect would take on faster and it was just curiosity I suppose. When I was experimenting with that I would swallow one pill whole and then while that was I guess digesting I would crush and snort a different pill • The immediate effect was definitely the reason. The effects overall were shorter lasting but more powerful in that method of use. I think so that was probably the main reason the immediacy of the effect of the drug • About 10–20 min faster than swallowing. I mean the oral takes about 10–20 min more than snorting it. Yes. And you when you snort it it's about 5, 5–10 min • Oh, absolutely I mean you know it hit you in the face like a truck and you feel like the most productive worker in the world. You can get up and you can conquer anything you want it makes you feel empowered if that makes sense
**Curiosity/Others were doing it/A friend was doing it**
• I was kind of introduced to it for the most part. And like you get that drip on the back of your throat when you do it. The constant reminder kind of higher by the availability and a lot of other methods for quick acting methods and so the constant taste is kind of a reminder, I guess • So, generally with friends it's a little more of a party thing. You know they all wanted to snort it, and nobody wanted to eat it just because it's cool to snort drugs or whatever. And so that's usually how it would go down. When I was in the company of others • I was dating a girl that had it and was doing it and I decided to try it
**Liked it and thought it was cool**
• It was cool to snort • Pretty much snorting. I mean I did other stuff like coke and stuff like that way before. So, I was kind of already in that mindset
**INTRAVENOUS**
**Faster impact (Intravenous)**
• I mean I told you earlier snorting it take maybe a few minutes and then you go on and you feel kind of good. But when you inject, it immediately hits. I mean as soon as you plunge the plunger down it immediately hits, and you get hit stronger and harder than any other way would hit
**Ritual**
• … As my addiction progressed, I wouldn't do anything if I couldn't shoot it. Basically • And through it all I feel like I was just as addicted to the using and the like the ritual of preparing the injection and all that just as much. You know it was just part of it
**Curiosity (Intravenous)**
• That was in part sheer dumb curiosity and reports I've read from the Internet. A lot of people on the Internet dangerously overhype what that's like. And you know it's very tempting once you're that far in: where you're already snorting and you're already taking it every day to say, well … how much more harmful than the next step be. And it is that much harmful because it is it's you know I don't want to say great but it's really great when you do that. ….*(sic)*

Eight participants (34.8%) said that prescription stimulant NMU influenced their subsequent use of illicit drugs. While there were numerous examples of illicit substances (primarily marijuana) influencing other illicit substance use, thirteen respondents (56.5%) indicated that use of illicit substances influenced their decision to try prescription stimulant NMU. Four participants (17.4%) reported that their initial prescription stimulant NMU affected their use of other prescription stimulants. Table 5 summarizes representative statements of these influential relationships. Supplementary Tables present the actual pathways among various substances reported by participants. Slightly more than half (13/23; 56.5%) reported using cocaine after using prescription stimulants, which is a transition within the stimulant class in addition to ongoing polydrug use.

**Table 5:** Examples of influences associated with prescription stimulant NMU among interview respondents (*n* = 23).

**Influence of prescription stimulant NMU on illicit substance use**
• Oh of course it did. Because it has opened up a world to me of not feeling pain. So, of course I was open to the idea of trying to have the feeling that this thing worked so well that I might as well see what else is out there • Experience with *methylphenidate product* influenced me to try other ‘speedy sort of drugs’, in particular cocaine • *Methylphenidate product* was a substitute for cocaine high • *Amphetamine product* helped with studying and cramming purposes, partying, enhancing marijuana and alcohol intake, could drink more and party longer
**Influence of illicit substances on prescription stimulant NMU**
• When (I) couldn't get cocaine (I) would inject prescription stimulants to try and chase that “cocaine high” • The biggest influence for me to start snorting …was my marijuana use was extreme. I was using the *amphetamine product* to enhance that and get a little bit higher from it. And this all probably played into it as well. But I was having severe depression symptoms and I was kind that was a coping mechanism although temporary
**Influence of prescription stimulant NMU on other prescription stimulant NMU**
• Positive experience with *amphetamine products* led me to be curious/try other prescription stimulants/fun habit/Positive experience with *long-acting amphetamine products* led me to try other time release stimulants • Curious to see how it (*methylphenidate product*) felt compared to *amphetamine product* • Wanted to find something to avoid sundowning/effect of *amphetamine product* wearing off • Crushed them and snorted or crushed them and ate them….because I was cramming for a test. Extra schoolwork • I was looking for an effect similar to *amphetamine product* and I was looking for sort of a potent something to keep me awake. It's something to keep me focused while I was doing work and that's why I snorted it instead of swallowing it which I felt like would be a less potent dose • Experience with *amphetamine product* influenced the use of other prescription stimulants as a result of *amphetamine product* getting too expensive for regular use • *Amphetamine product* made more comfortable taking prescription stimulants. So, if preference wasn't available, felt pretty comfortable going to another prescription stimulant • Well yes I did. I suppose if anything it made me more want to acquire *amphetamine product* to keep with that and have a more consistent supply because I saw the value of it both for academically and for partying

## Discussion and Conclusion

The purpose of this investigation was to characterize prescription stimulant NMU class, route of administration and substance transitions. A unique, convenience sample of adults who reported non-oral prescription stimulant NMU within the last 5 years were recruited from Reddit. Qualitative interviews among a subsample provided an opportunity to examine transitions in greater detail. This work reveals a gap not only in the academic literature, but also in general healthcare where prescription stimulant NMU may not be recognized (67).

### Illicit Substance Transitions

Almost 77% of the sample initiated an illicit substance use trajectory with marijuana during the ages that typically correspond to sophmore and junior years in high school. National Monitoring the Future surveys have found between 60 and 80% of high school sophmores report that marijuana has been “fairly easy” or “very easy” to obtain since 1992 (68), thus, this finding is not particularly surprising. Recent SAMSHA data indicating that 34.8% of adults from ages 18–25 years of age and 13.3% of adults 26 and older reported using marijuana in the past year further support this finding (2).

Almost 20% of the sample initiated their illicit substance use trajectory with prescription stimulant NMU. Three of those individuals only reported prescription stimulant NMU and did not transition to another illicit substance. The remainder primarily transitioned to marijuana. Marijuana remains a Schedule 1 substance and is illegal at the federal level, however, state-level laws vary with regard to use of medical marijuana, recreational use of marijuana and decriminalization, suggesting varying levels of ongoing access and use across the USA; its ubiquitousness is reflected in this sample.

While prescription stimulants were not always the first substance of the use trajectory, they were also not the last. Most illicit substance use occurred after the initial prescription stimulant NMU. Slightly more than one-third (34.8%) of participants in the qualitative study claimed that prescription stimulant NMU influenced their subsequent use of illicit substances, while almost one-half endorsed prescription stimulant NMU when their regular dose was not achieving its effect, a noted risk factor for misuse (69). Recently, among multiple cohorts of high school seniors who were followed longitudinally, it was demonstrated that any reported prescription stimulant misuse (compared to none or rare misuse) was more strongly associated with subsequent substance use disorder symptoms at age 35, including cannabis use disorder, other substance use disorder or any substance use disorder (70).

Prescription stimulants have also been found to be predictive factors in the development of illicit opioid use (48, 71) and increased concurrent use of opioids and stimulants has been represented in overdose deaths (72, 73), revealing a previously underappreciated level of risk associated with this particular trajectory. This finding is particularly important, given the recognition that many college-age students believe the level of risk associated with prescription stimulant NMU is low to non-existent (74).

### Route of Administration Transitions

Participants were recruited because they had used prescription stimulants non-orally, a risk factor for substance use severity (57, 66). For example, the speed of transition from first use to daily heroin was faster if the initial use was non-oral (injection) (58). Less is known and reported about the transitions from oral to non-oral prescription stimulant ROAs, other than they are likely to occur during the college years (1, 41, 60), and that they are likely to occur (26–28).

The present study found that the majority of the sample reported oral NMU prior to non-oral NMU. Most, after oral use, transitioned to snorting prescription stimulants (~70%), or only ever snorted prescription stimulants (23%). These data add to recent findings that most non-oral use of prescription stimulants is intranasal among adults (26–28) and adolescents (19, 41).

However, among some, ROA moved beyond intranasal use to include smoking and injection. Intranasal and injection prescription stimulant NMU are associated with more severe medical outcomes than oral NMU (75), and thus awareness of these potential ROA transitions in prescription stimulant NMU is critical. For example, multiple, non-linear routes of administration have been reported for prescription opioid NMU (76) where approximately half of those interviewed transitioned to snorting or injection of prescription opioids before entering treatment. Similar to prescription stimulant ROA transitions, opioid ROA transitions were mostly to achieve a desired effect or due to social influences, where social influences typically led them to undertake a more dangerous ROA (76). Similar relationships may exist among prescrtipion stimulants, which is an avenue for future investigation.

### Class Transitions

The present study found evidence of stimulant class transitions whereby (in addition to most illicit substance use), most illicit stimulant use occurred after prescription stimulant NMU. There is little to no available data on this specific transition. It has been found that early prescription drug NMU is associated with later prescription drug abuse and dependence (77), and prescription stimulant use is associated with subsequent substance use or dependence (24, 78). Similarly, the class transition from prescription to illicit substance has been noted with opioids where prescription opioids have pre-dated the use of heroin (51, 53, 79). When the class transition was made to an illicit stimulant, cocaine, it was snorted, and in 12/2/3 (52%) of those interviewed, this occurred after previous prescription stimulants had been tampered with and/or snorted. Of 16.7% of those with lifetime cocaine use and 11.2% of those with lifetime amphetamine use go on to develop stimulant use disorders (80), and it is possible that the present data capture the initial stages of those trajectories.

### Limitations

Data were obtained from a convenience sample of adults who were recruited from Reddit. Although Reddit is advertised as the 5th most visited website in the United States, this may not be a representative sample of adults who undertake non-oral prescription stimulant NMU. Reddit users have been characterized as young, male, regular internet users (81). Subsequent studies targeting non-oral prescription stimulant NMU are needed; the present study can inform hypothesis generation. Increasing attention is being paid to the use of Reddit specifically (82–87) for the conduct of substance use research. One distinct advantage of social media platforms is the potential reach to hidden populations, such as substance users (88). Although data are self-reported, given the nature of the information disclosed, it is possible that anonymous online surveys may increase the likelihood of truthful reporting. In the present study, the self-reported information collected in the quantitative survey was verified and detailed among those who participated in the follow-up interviews.

Temporal relationships alone cannot determine causality. While a mixed methods approach was used to capture motivation and factors that influenced trajectories of substance use, we are unable to quantify the causal relationship between prescription stimulant NMU (or non-oral prescription stimulant NMU) and subsequent drug use patterns. Quantitative data measure study outcomes with precise, numerical data that can be generalized and compared with statistical tests that are widely recognized (89). However, such data can also be overly general and, as a result, decontextualize the environment in which the outcomes of interest (in this case, substance use behaviors) take place (90). They may not convey the “lived experience” (91). Qualitative data, where the word is the unit of analysis, are employed to examine experiences or processes and can convey meaning and subtlety in a way that numbers alone cannot (90). The mixed method strategy, including quantitative and qualitative data collected together (92) allows for a more nuanced interpretation of each type of data (93) and as such, is a design choice that is particularly useful in the study of non-medical substance use behaviors (74). These behaviors are not only difficult to quantify, but are stigmatized and often difficult to find and detail in the general population, rendering mixed methodology a useful and valuable data collection strategy. In the present study, this strategy enabled a rich characterization of transitions and use pathways.

### Conclusion and Future Directions

This study found prescription stimulant NMU to be associated with substance transitions, route of administration transitions and class transitions, which is a novel clarification of the risks associated with this type of substance use. This study was undertaken before the FDA issued the Register notice [FDA-2019-N-3403; Federal Register 84 (183), September 20, 2019], however, some of the findings may inform the questions raised in that document, specifically, whether there may be a role for manipulation resistant formulations in the current misuse and abuse of prescription stimulants. While illicit stimulants are not data presented herein may be used to inform future studies that address whether interventions, such as manipulation resistance formulations of prescription stimulant medications, may disrupt the key transitions that are part of substance use trajectories.

## Data Availability Statement

Data are proprietary. Requests to access the data may be considered.

## Ethics Statement

The studies involving human participants were reviewed and approved by New England Institutional Review Board. The patients/participants provided their written informed consent to participate in this study.

## Author Contributions

SV evaluated early data analyses, suggested subsequent data analyses, wrote the first draft, and revised the manuscript. RR suggested and conducted data analyses and reviewed the manuscript. KA and SF reviewed the manuscript and made significant helpful suggestions and comments. JG acquired funding, designed and oversaw the study, suggested data analyses, and reviewed and commented on the manuscript extensively. All authors contributed to the article and approved the submitted version.

## Conflict of Interest

RR and JG are employees of Inflexxion, an IBH Company. SV is an independent scientific writer who contracts with Inflexxion. Inflexxion contracts with FDA and multiple companies with interests in some of the products included in the compounds evaluated for this article. Although the sponsor was involved in reviewing the content of this article, all data collection, analysis, and ultimate data interpretation were made by the authors without sponsor influence. KA serves as an Advisor to Arbor Pharmaceutical Company and receives research funding from Takeda Pharmaceutical Company. In the past year, SF received income, potential income, travel expenses continuing education support, and/or research support from Takeda, OnDosis, Tris, Otsuka, Arbor, Ironshore, Rhodes, Akili Interactive Labs, Sunovion, Supernus, and Genomind. With his institution, he has US patent US20130217707 A1 for the use of sodium-hydrogen exchange inhibitors in the treatment of ADHD, receives royalties from books published by Guilford Press: *Straight Talk about Your Child's Mental Health*, Oxford University Press: *Schizophrenia: The Facts* and Elsevier: ADHD: *Non-Pharmacologic Interventions*, and also Program Director of www.adhdinadults.com.
