# Supplementary material for: Characterizing Pathways of Non-oral Prescription Stimulant Non-medical Use Among Adults Recruited From Reddit
Source: Front Psychiatry. 2021 Jan 25;11:631792. doi: 10.3389/fpsyt.2020.631792 (PMC7883730; doi:10.3389/fpsyt.2020.631792)
Supplement: Supplementary file 1 [file Table_1.DOCX]

## Supplemental Tables

**Participant chronologies across of use of substances by age (years) and route of administration**

**Substances and routes of administration are color coded.**

Participant 1 (20 years old at time of interview).

| Ritalin | Vyvanse | Other prescription stimulant | Marijuana | Ritalin LA | Focalin | Focalin XR | Amphetamines | Cocaine  or crack | Hallucinogens |
| --- | --- | --- | --- | --- | --- | --- | --- | --- | --- |
| 15  Swallow | 15  Swallow | 15  Swallow | 16  Smoke | 17  Snort | 17 Swallow | 17  Swallow | 18  Swallow | 19  Snort | 19 Chew/dissolve |
|  | 18  Snort | 18  Snort |  |  | 18  Snort | 18  Snort |  |  |  |

Participant 2 (24 years old at time of interview).

| Marijuana | Cocaine or crack | Hallucinogens | Adderall | Amphetamines |
| --- | --- | --- | --- | --- |
| 14  Smoke | 17  Snort | 18  Chew/dissolve | 18 or 19  Swallow | 24  Swallow |
|  |  |  | 18 or 19  Snort |  |

Participant 3 (19 years old at time of interview).

| Focalin | Marijuana | Adderall | Adderall XR | Hallucinogens | Vyvanse |
| --- | --- | --- | --- | --- | --- |
| 17  Swallow | 18  Chew/dissolve | 18  Swallow | 18  Swallow | 18 yrs.  Chew/dissolve | 18  Swallow |
|  | 18  Smoke | 18  Dissolve liquid | 18  Dissolve liquid |  | 18  Dissolve liquid |
|  |  | 18  Snort |  |  |  |

Participant 4 (21 years old at time of interview).

| Marijuana | Hallucinogens | Amphetamines  [MDMA] | Adderall | Other Illicit Substances | Adderall XR | Concerta | Ritalin | Vyvanse | Cocaine | Methamphetamine | Barbiturates |
| --- | --- | --- | --- | --- | --- | --- | --- | --- | --- | --- | --- |
| 16  Smoke | 17 (Mushrooms)  Chew/dissolve | 18  Chew/dissolve | 18  Swallow | 18  Swallow | 19  Swallow | 19  Swallow | 19  Swallow | 19  Swallow | 19  Snort | 20  Swallow | 21  Swallow |
|  | 18 (LSD)  Chew/dissolve | 18  Smoke | 19  Snort | 20  Inject | 19  Snort | 19  Snort |  |  |  | 21  Smoke |  |
|  |  |  | 20  Inject |  |  |  |  |  |  | 21  Inject |  |

Participant 5 (33 years old at time of interview).

| Marijuana | Adderall | Adderall XR | Inhalants | Hallucinogens | Cocaine | Crack | Ritalin LA | Ritalin SR | Ritalin | Other illicit substances | Heroin | Street fentanyl |
| --- | --- | --- | --- | --- | --- | --- | --- | --- | --- | --- | --- | --- |
| 12  Smoke | 14  Snort | 14  Snort | 16  Inhale | 18  Chew/dissolve | 18  Snort | 18  Smoke | 25  Inject | 25  Inject | 25  Inject | 25  Inject | 27  Inject | 28  Inject |
|  |  |  |  |  | 25  Inject | 25  Inject |  |  |  |  |  |  |

Participant 6 (23 years old at time of interview).

| Marijuana | Inhalants | Adderall XR | Adderall | Ritalin LA | Ritalin  Dextrostat  Vyvanse | Focalin XR  Concerta | Hallucinogens | Cocaine | Meth | Heroin | Barbiturates | Crack |
| --- | --- | --- | --- | --- | --- | --- | --- | --- | --- | --- | --- | --- |
| 13  Smoke | 14  Inhale | 14  Swallow | 14  Swallow | 14  Swallow | 14-15  Swallow | 15  Swallow | 15  Swallow | 15  Snort | 16  Swallow | 16  Smoke | 17  Swallow | 18  Smoke |
|  |  | 14  Chew/dissolve | 14  Chew/dissolve | 15  Chew/dissolve | 14-15  Chew/dissolve | 15  Snort |  |  | 16  Chew/dissolve | 18  Inject | 17  Dissolve liquid |  |
|  |  | 14  Dissolve liquid | 17  Snort | 15  Snort | 14-15  Snort |  |  |  | 16  Snort |  |  |  |
|  |  | 17  Smoke |  |  | 15-17  Smoke |  |  |  | 16  Smoke |  |  |  |
|  |  |  |  |  |  |  |  |  | 17-18  Inject |  |  |  |

Participant 7 (33 years old at time of interview).

| Marijuana | Adderall | Adderall XR | Cocaine | Ritalin LA | Methylin ER | Concerta |
| --- | --- | --- | --- | --- | --- | --- |
| 15-16  Smoke | 18  Swallow | 18  Swallow | 19  Snort | 31  Swallow | 31  Swallow | 31  Swallow |
| 33  Chew/dissolve | 18  Chew/dissolve |  |  |  |  |  |

Participant 9 (27 years old at time of interview).

| Marijuana | Hallucinogens | Amphetamines  [MDMA] | Cocaine | Adderall XR | Adderall |
| --- | --- | --- | --- | --- | --- |
| 16  Smoke | 20  Chew/dissolve | 20  Swallow | 21  Snort | 22  Swallow | 22  Swallow |
|  |  |  |  |  | 22  Chew/dissolve |
|  |  |  |  |  | 23  Snort |

Participant 10 (37 years old at time of interview).

| Ritalin | Ritalin LA | Marijuana | Adderall XR | Methylin | Hallucinogens | Cocaine | Concerta | Vyvanse |
| --- | --- | --- | --- | --- | --- | --- | --- | --- |
| 18  Swallow | 18  Dissolve liquid | 19  Smoke | 19  Chew/dissolve | 19  Swallow | 21  Swallow | 21  Snort | 31  Swallow | 32  Dissolve liquid |
| 18  Snort | 18  Snort |  | 19  Dissolve liquid |  |  |  | 31  Cut or broke |  |

Participant 11 (36 years old at time of interview).

Participant 11 (Chronology of drug use by age and route)

| Marijuana | Hallucinogens | Cocaine | Inhalants | Street fentanyl | Adderall | Methylin |
| --- | --- | --- | --- | --- | --- | --- |
| 14  Smoke | 18  Swallow | 21  Snort | 22  Inhale | 25  Dissolve liquid | 25  Swallow | 33  Smoke |
|  | 20  Dissolve liquid |  |  |  | 25  Cut or broke |  |
|  |  |  |  |  | 30  Snort |  |

Participant 13 (31 years old at time of interview).

Participant 11 (Chronology of drug use by age and route)

| Marijuana | Adderall | Adderall XR | Inhalants | Hallucinogens | Other illicit substance |
| --- | --- | --- | --- | --- | --- |
| 18  Smoke | 19  Swallow | 19  Swallow | 19  Inhale | 20  Chew/dissolve | 21  Snort |
| 18  Chew/dissolve | 31  Snort |  |  |  |  |

Participant 14 (28 years old at time of interview).

Participant 11 (Chronology of drug use by age and route)

| Marijuana | Hallucinogens | Adderall |
| --- | --- | --- |
| 19  Smoke | 22  Swallow | 24  Swallow |
| 22  Chew/dissolve |  | 25  Snort |

Participant 15 (27 years old at time of interview).

Participant 11 (Chronology of drug use by age and route)

| Marijuana | Vyvanse | Cocaine | Adderall | Dexedrine | Amphetamines  [meth + Adderall pressed pill] | Hallucinogens | Adderall XR |
| --- | --- | --- | --- | --- | --- | --- | --- |
| 18  Smoke | 19  Swallow | 20  Snort | 20  Swallow | 20  Swallow | 20  Swallow | 24  Swallow | 24 or 25  Snort |

Participant 16 (27 years old at time of interview).

| Marijuana | Adderall XR | Concerta | Hallucinogens | Amphetamines  [MDMA] | Adderall | Vyvanse | Cocaine | Other illicit substances | Inhalants |
| --- | --- | --- | --- | --- | --- | --- | --- | --- | --- |
| 17  Smoke | 17  Swallow | 17  Swallow | 17  Swallow | 17  Swallow | 19  Swallow | 19  Swallow | 19  Snort | 25  Swallow PCP | 27  Inhale |
| 17  Chew/dissolve | 19  Dissolve liquid | 17  Chew/dissolve | 17  Dissolve liquid | 18  Snort | 19  Chew/dissolve |  |  | 27  Smoke Opium |  |
|  | 19  Snort |  |  |  | 19  Dissolve liquid |  |  |  |  |
|  |  |  |  |  | 19  Snort |  |  |  |  |
|  |  |  |  |  | 26  Cut or broke |  |  |  |  |

Participant 17 (28 years old at time of interview).

| Marijuana | Methamphetamines | Dexedrine  Dexedrine spansules | Dextrostat | Crack | Inhalants | Adderall XR  Desoxyn | Vyvanse | Cocaine | Adderall | Hallucinogens | Heroin | Ritalin SR  Ritalin  Ritalin LA |
| --- | --- | --- | --- | --- | --- | --- | --- | --- | --- | --- | --- | --- |
| 15  Smoke | 16  Smoke | 22  Swallow | 22  Chew/dissolve | 22  Snort | 22  Inhale | 22  Chew/dissolve | 22  Swallow | 22  Snort | 22  Swallow | 22  Smoke | 22  Smoke | 22  Cut or broke |
|  | 16  Snort |  |  |  |  |  | 22  Dissolve liquid |  | 22  Snort | 22  Chew/dissolve | 22  Snort | 22  Snort |
|  | 16  Inject |  |  |  |  |  | 22  Snort |  | 22  Inject |  | 22  Inject | 22  Inject |

Participant 18 (32 years old at time of interview).

| Cocaine | Marijuana | Barbiturates | Hallucinogens | Inhalants | Adderall | Amphetamines  [uppers} | Ritalin  Dexedrine | Adderall XR  Vyvanse | Heroin | Street fentanyl | Other illicit substances* |
| --- | --- | --- | --- | --- | --- | --- | --- | --- | --- | --- | --- |
| 16  Snort | 17  Smoke | 16  Snort | 16  Swallow | 17  Inhale | 17  Swallow | 17  Swallow | 18  Swallow | 20  Snort | 20  Smoked | 22  Smoke | 24  Snort |
| 16 -18  Smoke |  |  |  |  | 17  Chew/dissolve |  |  |  | 20  snort |  |  |
|  |  |  |  |  | 17  Snort |  |  |  |  |  |  |

Participant 19 (20 years old at time of interview).

Participant 11 (Chronology of drug use by age and route)

| Marijuana | Adderall | Hallucinogens |
| --- | --- | --- |
| 17  Smoke | 18  Swallow | 19  Chew/dissolve |
| 18  Dissolve liquid | 18  Snort |  |
| 19  Chew/dissolve |  |  |

Participant 20 (36 years old at time of interview).

Participant 11 (Chronology of drug use by age and route)

| Marijuana | Ritalin | Other illicit substances | Hallucinogens | Inhalants | Amphetamines  [MDMA] | Cocaine | Crack | Barbiturates | Adderall XR | Methamphetamine | Heroin |
| --- | --- | --- | --- | --- | --- | --- | --- | --- | --- | --- | --- |
| 14  Smoke | 14  Snort | 14  Snort | 15 (Blotter acid)  Chew/dissolve | 16 or 17  Inhale | 19  Snort | 19  Snort | 20  Smoke | 22  Snort | 35  Snort | 35  Snort | 35  Snort |
| 20  Chew/dissolve | 15  Swallow |  | 19 (LSD, Mushrooms)  Chew/dissolve |  |  |  |  |  |  | 35  Smoke  “hot railing” |  |

Participant 21 (35 years old at time of interview).

Participant 11 (Chronology of drug use by age and route)

| Ritalin | Marijuana | Dexedrine spansules | Hallucinogens | Adderall | Vyvanse | Cocaine | Heroin | Other illicit substances | Inhalants | Crack | Street fentanyl |
| --- | --- | --- | --- | --- | --- | --- | --- | --- | --- | --- | --- |
| 9  Swallow | 15  Smoke | 16  Snort | 18 (Mushrooms)  Chew/dissolve | 19  Cut or broke | 19  Snort | 20  Snort | 23  Smoke | 23  Snort | 23  Inhale | 29  Smoke | 31  Smoke |
|  |  |  | 21 (LSD)  Chew/dissolve | 19  Snort |  |  | 25  Snort | 23  Smoke |  |  | 32  Inject |
|  |  |  |  |  |  |  |  |  |  |  |  |

Participant 22 (29 years old at time of interview).

Participant 11 (Chronology of drug use by age and route)

| Marijuana | Hallucinogens | Amphetamines  [MDMA] | Cocaine | Adderall XR | Concerta | Other illicit substances | Inhalants | Adderall | Heroin | Barbiturates | Crack | Methamphetamine | Street fentanyl |
| --- | --- | --- | --- | --- | --- | --- | --- | --- | --- | --- | --- | --- | --- |
| 14  Smoke | 15  Chew/dissolve | 15  Chew/dissolve | 15  Snort | 15  Swallow | 15  Swallow | 15  Swallow | 16  Inhale | 16  Swallow | 17  Smoke | 20  Swallow | 20  Smoke | 20  Smoke | 22  Other route [nasal spray. Patch] |
|  |  | 16  Dissolve liquid |  | 15  Cut or broke |  | 15  Snort |  | 16  Cut or broke | 18  Snort | 20  Snort |  | 24  Snort |  |
|  |  |  |  | 15  Chew/dissolve |  |  |  | 16  Chew/dissolve |  |  |  |  |  |
|  |  |  |  | 15  Snort |  |  |  | 16  Snort |  |  |  |  |  |

Participant 23 (19 years old at time of interview).

Participant 11 (Chronology of drug use by age and route)

| Marijuana | Hallucinogens | Amphetamines  [MDMA] | Focalin XR | Inhalants |
| --- | --- | --- | --- | --- |
| 18  Smoke | 18  Swallow | 18  Chew/dissolve | 18  Snort | 19  Inhale |
|  | 18  Chew/dissolve |  |  |  |

Participant 24 (22 years old at time of interview).

Participant 11 (Chronology of drug use by age and route)

| Ritalin | Marijuana | Hallucinogens | Concerta | Adderall | Adderall XR  Vyvanse | Cocaine | Amphetamines  [amph powder] | Focalin XR | Focalin |
| --- | --- | --- | --- | --- | --- | --- | --- | --- | --- |
| 14  Swallow | 14  Smoke | 19  Swallow | 19  Swallow | 19  Swallow | 19 - 20  Swallow | 19 - 20  Snort | 19 - 20  Snort | 20  Swallow | 20  Swallow |
| 14  Snort | 18  Chew/dissolve |  |  | 20  Snort | 19 - 20  Chew/dissolve |  |  | 20  Chew/dissolve | 20  Snort |
|  |  |  |  |  |  |  |  |  | 20  Chew/dissolve |

Participant 25 (33 years old at time of interview).

| Barbiturates | Adderall XR | Marijuana | Ritalin  Adderall | Ritalin SR | Hallucin-ogens | Inhalants | Desoxyn | Barbiturates | Other illicit substances | Cocaine | Heroin | Methamphet-amine |
| --- | --- | --- | --- | --- | --- | --- | --- | --- | --- | --- | --- | --- |
| 11  Swallow | 12  Swallow | 13  Smoke | 13  Swallow | 15  Swallow | 15  Swallow | 16  Inhale | 19  Swallow | 19  Swallow | 19  Swallow | 23  Snort | 27  Snort | 30  Swallow |
|  |  | 15  Swallow | 21  Cut or broke | 21  Cut or broke |  |  |  | 19  Chew/dissolve | 19  Chew/dissolve |  |  | 30  Chew/ dissolve |
|  |  | 15  Chew/dissolve | 22  Chew/dissolve | 22  Chew/dissolve |  |  |  | 19  Snort | 19  Snort |  |  | 30  Other route  “booty bump” |
|  |  |  | 23  Snort | 23  Snort |  |  |  |  |  |  |  |  |
